# How do we perform backward serial recall?

**DOI:** 10.3758/s13421-018-0889-2

**Published:** 2019-02-15

**Authors:** Dennis Norris, Jane Hall, Susan E. Gathercole

**Affiliations:** MRC Cognition and Brain Sciences Unit, University of Cambridge, 15 Chaucer Road, Cambridge, CB2 7EF UK

**Keywords:** Memory, Short-term memory, Serial recall

## Abstract

Following Conrad (1965, *Journal of Verbal Learning and Verbal Behavior, 4*, 161–169) it is often assumed that backward verbal serial recall is performed by repeated forward scans through the list and then recalling the last remaining item. Direct evidence for this peel-off strategy is relatively weak, and there has to date been no examination of its potential role in the recall of spatial sequences. To examine the role of this strategy in both verbal and spatial domains, two experiments examined response output times for forward and backward recall. For spatial span, the pattern of timing was the same in both directions. For digit span, backward recall was considerably slower. This was true whether responses were made by means of manual selection on a keyboard display (Experiment 1) or were spoken (Experiment 2a). Only two of 24 participants showed signs of using a peel-off strategy in spoken backward recall. Peel-off was not a dominant strategy in backward digit recall and there was no indication that it was ever used for spatial stimuli. Most participants reported using a combination of different strategies. In Experiment 2b, four further participants were directly instructed to use a peel-off strategy. The pattern of response times for three of these individuals was similar to the two participants from Experiment 2a previously identified as using peel-off. We conclude that backward recall can be performed using many strategies, but that the peel-off is rarely used spontaneously.

The ability to remember spoken items in the order in which they were presented is critical to understanding spoken language, remembering telephone numbers, PIN numbers, passwords, and instructions, as well as to learning new words (Baddeley et al., 1998). In everyday life, circumstances rarely arise that require sequences to be reversed. Despite this low ecological validity, backward recall is something that we can do, and understanding how we do it may have important implications for the broader field of learning. For many decades, backward digit span has occupied a prominent position through its inclusion in test instruments extensively used in psychological research and neuropsychological evaluation (Elliott, Murray, & Pearson, 1990; Wechsler, 2014). It is one of the most reliable measures of general and complex cognitive abilities, predicting children’s current and future academic learning (Bull, Espy, & Wiebe, 2008; Gathercole, Pickering, Knight, & Stegmann, 2004) and showing high sensitivity to age-related cognitive decline (Bopp & Verhaeghen, 2005). So, just how do we recall in backward order, and how might this relate to individual differences in broader cognitive function?

Some facts about backward recall are already known (for review, see Donolato, Giofrè, & Mammarella, 2017). It is usually less accurate than forward recall (e.g., Anders & Lillyquist, 1971). The most commonly held view is that it is performed by a series of forward recalls (Anders & Lillyquist, 1971; Anderson, Bothell, Lebiere, & Matessa, 1998; Conrad, 1965; Murdock, 1995; Page & Norris, 1998; Thomas, Milner, & Haberlandt, 2003). Conrad (1965) stated that “within the memory span, a succession of rapid to-and-fro scans would be adequate to simulate backward recall” (p. 169). The implication is that the underlying representation is intrinsically forward. Other evidence suggests that visuospatial imagery may be used to support backward recall (Hoshi et al., 2000; Li & Lewandowsky, 1995) and that, in typical adults, at least, this depends the same verbal STM (short-term memory) system as forward span (St Clair-Thompson & Allen, 2013).

Although recall is usually poorer in backward than in forward order, several studies have failed to find an overall difference in accuracy between forward and backward recall (Anderson et al., 1998; Bireta et al., 2010; Farrand & Jones, 1996; Li & Lewandowsky, 1995; Thomas et al., 2003), and others have shown superior performance for backward recall (Guérard, Saint-Aubin, Burns, & Chamberland, 2012, Experiment 4a, Experiment 5; Madigan, 1971). Hurlstone, Hitch, and Baddeley (2014) suggested that the critical factor may be testing procedure: Whereas span tasks typically show a forward advantage, fixed list-length tasks are less likely to do so. Qualitative differences between the two paradigms have often been reported. Bireta et al. (2010), for example, found that each of the characteristic effects of word length, irrelevant speech, phonological similarity, and concurrent articulation in forward verbal serial recall was eliminated with backward recall.

In contrast, differences in the accuracy of forward and backward spatial recall have rarely been reported (Isaacs & Vargha-Khadem, 1989; Vandierendonck, Kemps, Fastame, & Szmalec, 2004; Wilde & Strauss, 2002). This equivalence implies that the representation of order can be interrogated equally well in either direction. It could either be a consequence of the nature of the representations or of the retrieval processes developed to act upon these. There may be nothing intrinsically directional about the representations themselves, in contrast to representations of verbal serial order, which appear to be preferentially configured for the forward-going retrieval of order.

A peel-off strategy should be evident in the timing of output responses. If backward recall is achieved by peeling off items in successive forward retrievals, output time should be a negatively accelerated function across serial position. This is because output of the last list item in an *N*-item list will require *N* items to be retrieved from memory, the next item will require *N* − 1 items to be retrieved, and so forth, for sequences of diminishing length. In contrast, the simplest model assumes that retrieval can operate with equal facility in either direction and that output time will be a linear function of recall position for both forward and backward recall. This prediction was first tested directly by Anders and Lillyquist (1971) in an experiment that timed the spoken forward and backward recall of digits. They concluded that participants do peel off, reporting “retrieving the last two or three items of the list as a group, reading them out in backward order, dipping back into memory, retrieving the next group of two or three items, reading them out in backward order, and so forth until recall was completed” (p. 206).

Although consistent with participants’ strategy reports, the response time data offered by Anders and Lillyquist (1971) in support of this position is weak at best. Their primary measure was cumulative interdigit pause time. Pause time was a linear function of output position, with steeper slopes for backward than for forward recall. It was noted that the duration of the spoken digits was greater for backward than for forward recall, “reflecting, perhaps, the Ss’ habit of drawing out sounds to fill delays in speech and maintain the continuity of their responses” (p. 206). It may therefore have been more informative to report and analyze the onset time of each spoken digit, eliminating the variability in the actual production of the spoken item. Other details of the study were not clearly specified. Although the list length was reported as being set at each participant’s span, the data were described as being averaged to produce a single summary plot of output times by serial position with eight items. However, we know that not all participants had a span of eight because Anders and Lillyquist presented illustrative data for one participant with a span of six.

Haberlandt, Lawrence, Krohn, Bower, and Thomas (2005) measured spoken forward and backward recall of words chosen from an open set at list lengths of four, five, and six. Backward recall was slower than forward recall. In this study, the timing data were not used to draw inferences about how backward recall was accomplished and it was assumed that participants used the peel-off strategy. Furthermore, the data were relatively sparse. Timing data were reported for all items in the correct position, regardless of any errors on other items in the list. There were only 12 trials at each list length, data were from all participants who got at least one item correct at each list position for each list length. A participant might therefore have contributed only a single data point from a list in which they correctly recalled only one item.

Guerrette, Guérard, and Saint-Aubin (2017) measured forward and backward spoken recall of auditorily presented nonsense syllables in the context of a Hebb repetition task (Hebb, 1961). They reported that the rate of output did not increase across output position, as would be expected by the peel-off strategy. Instead, they suggested that recall might be achieved by reversing the order of items within groups of stimuli.

Four other studies to our knowledge also examined timing of forward and backward recall, although none used spoken recall. More convincing evidence that backward recall might be achieved by successive forward scans comes from Anderson et al. (1998). Their study used lists of different lengths. Although participants were tested on either grouped or ungrouped lists, the data were reported only for participants with grouped lists. Recall was typed. Anderson et al. simulated their data using the ACT-R model under the assumption that backward recall was performed by successive forward recalls of groups, with items in a group becoming available simultaneously. The model provided a good fit to the data, although the corresponding outcome with ungrouped lists is not reported.

Thomas et al. (2003) used lists of words and had participants type their responses. Interresponse-onset times were quite slow, at 2 to 4 seconds. Bireta et al. (2010) also used word lists, but had participants respond by clicking on each of the words in succession. Direction of recall was determined by a postcue, ensuring that the encoding was equivalent in each recall order condition. Recall accuracy was higher for forward than backward recall in some but not all of the four experiments reported. Surprenant et al. (2011a, 2011b) used a similar procedure to Bireta et al. (2010), but also manipulated the order in which forward and backward trials were presented and whether participants knew the direction of recall in advance. In both experiments, the rate of recall was faster for forward than for backward recall, but this difference was significant only in their Experiment 2.

With the exception of the figures presented in an unpublished report by Surprenant et al. (2011b), Supplemental Materials, previous studies have reported response-time functions averaged across participants. This obscures potential individual differences in the strategies adopted across individuals. Some of the experiments also have high error rates. This is a source of noise if the focus is on RTs, as response speed on error trials will include guesses, pauses where participants fail to retrieve an item, and error recovery processes.

In this study, we report data from two experiments examining whether response times in backward recall reflect a forward-going peel-off strategy. They were designed to overcome some of the limitations of the small number of previous relevant studies and also to test whether strategies for backward recall such as peel-off are restricted to verbal material or can be extended to the spatial domain. Conrad’s (1965) original proposal was that the peel-off strategy for backward recall is restricted to span-length lists. Our experimental trials therefore employed list lengths set to the individual spans of the participants, and data were analyzed only for lists that were correctly recalled.

Experiment 1 compared forward and backward recall of visually presented digit sequences and also of sequences of spatial locations. We know of no other data on the timing of backward recall in spatial short-term memory, and there are good grounds for suspecting that strategies to cope with backward recall may well be different across the verbal and spatial domains. We chose digits as our verbal stimuli for consistency with Anders and Lillyquist (1971). The fact that there is little, if any, recall cost to backward spatial recall (Isaacs & Vargha-Khadem, 1989; Wilde & Strauss, 2002) suggests that the spatial memory representations may be retrieved with equal ease in either direction. In contrast, backward recall of verbal sequences is frequently slow and errorful, in keeping with a time-consuming retrieval process such as successive forward scanning.

Such differences may not be too surprising given the distinctive functions that verbal and visuospatial STM may be designed to serve. In perception and comprehension of spoken (and, to some extent, printed) words, the input must be processed in a forward order. Memory for linguistic input should therefore maintain a representation in the same forward order. This applies not only at the level of words, but also to memory for sublexical segments such as phonemes. Spatial short-term memory serves a rather different purpose. It needs to be able to provide answers to questions such as “Where did that come from?” and “How do I move it back there?” as well as “Where is this going?”

In order to equate presentation and recall conditions as much as possible across the digit and spatial recall tasks, all stimuli were presented visually on an iPad, and responses were made by tapping on a virtual numeric keypad (telephone layout) in digit recall and on unfilled spatial locations in spatial recall. To anticipate, Experiment 1 provided little evidence that participants employ a peel-off strategy in backward recall of either digits or spatial locations. A possible explanation was that this strategy was not adopted in digit recall because the virtual keypad provided the opportunity for spatial recoding of verbal responses. This may have removed the necessity of a strategy of successive covert forward scans through verbal STM. To test this, Experiment 2 adopted the Anders and Lillyquist (1971) procedure of auditory presentation and spoken recall in both the forward and backward direction. Experiment 2b compared response times using the same procedure for participants both under conditions of no strategy instruction as in Experiment 2 and following direct instruction to use the peel-off strategy. Strategy report data were collected in all experiments. This allowed us to test directly the correspondence between the response time functions and reported strategy use.

As our primary focus was in identifying strategy use, the priority was to collect sufficient data to allow two contrasting mathematical models corresponding to peel-off and forward recall strategies to be fitted to the timing data from each participant. Backward serial recall studies often employ as 10 or fewer trials per condition (Bireta et al., 2010; Li & Lewandowsky, 1995; Surprenant et al., 2011a). The original Anders and Lillyquist (1971) timing experiment presented 76 trials in each condition, but from just 10 participants. In Experiment 1, 16 participants completed 80 trials in each recall direction in order to provide a better sample of the range of possible strategies.

## Experiment 1

### Method

Sixteen participants aged 18 to 35 years from the Medical Research Council Cognition and Brain Sciences Unit volunteer panel performed digit recall (mean age = 23.1 years, seven males), and a further 16 performed the spatial recall task (mean age = 23.6 years, five males). Both tasks were presented on an iPad with a display resolution of 2048 × 1536 pixels in landscape mode. In both Experiments 1 and 2, forward recall was performed in the first half of the experiment and backward recall in the second. This fixed order was employed to avoid possible carryover effects produced by practice in an unusual recall direction.

### Span pretest

For both tasks, participants first completed a span test. Span was determined by presenting blocks of six lists, starting with lists of two items. List length was increased by one if at least four lists were recalled completely correctly. Span was calculated the as the longest length where participants got at least four lists correct. The subsequent experimental phase employed lists that matched the individual’s span.

### Digit recall

Digits were presented at a rate of one per 750 ms, with each digit being displayed for 500 ms, with a blank interval of 250 ms between digits. At the end of the digit sequence, a numeric keyboard (digits 1–9 in telephone layout) was displayed, and participants had to recall the sequence by tapping the keys in the appropriate order. Below the keyboard there was a “Done” key that had to be pressed once recall had been completed. The time allowed to make a response was determined by the length of the list and was 7,500 ms for three-item lists plus 1,000 ms extra for every extra item.

With list lengths of nine or less, the digits were sampled randomly without replacement from the digits 1–9. For list lengths greater than nine, the initial set of nine digits was supplemented with a further randomly sampled *N* digits. Note that this was only necessary for setting the span, as no participant had a span of greater than nine (when testing span the list length could be 10, one digit would appear twice). No digits appeared twice in succession, and there were no runs of three or more consecutive ascending or descending digits. Participants performed 80 trials in the forward direction, followed by 80 in the backward direction.

### Spatial recall

The general procedure was the same as for digit recall. Participants were presented with an array of dark-blue circles on a gray background. Circles were 91 pixels in diameter and were randomly positioned over the entire screen subject to the constraint that they had a minimum center-to-center and center-to-edge separation of 272 pixels. The location of the circles was randomized afresh for each trial. The number of circles was the same as the list length. In random sequence, each circle turned light blue for 250 ms, followed by a pause of 500 ms before the next circle changed color. At the end of the sequence all circles remained visible and participants had to touch the circles in the designated order. As with digit recall, there were 80 trials in each recall direction, with forward recall being performed before backward recall.

### Strategy reports

At the end of the experiment participants completed a questionnaire related to each recall direction and were asked begin by reporting on the backward recall task they performed. The questionnaire listed 10 possible strategies (see Table 1). The strategy alternatives were based both on strategy descriptions relevant to forward and backward serial recall in the working memory questionnaire developed by Dunning and Holmes (2014), and from open reports of participants during pilot testing of both tasks. Participants were asked to rate each strategy in terms of the frequency with which they used it. The response options were *never*, *occasionally*, *frequently*, and *almost always*.

**Table 1:** Self-reported strategy use in Experiment 1 as a function of recall direction and stimuli

Recall direction:	Forward		Backward	
Strategy	Digits	Spatial	Digits	Spatial
Rehearse the items as they were presented	2.44	1.94	2.25	1.75
Group the items by separating them into sets of particular sizes	2.25	1.19	2.00	0.88
Group the items according to the pattern they form	0.50	2.13	0.69	1.63
Group the items according to their meaning	0.25	0.38	0.31	0.13
Form a mental image	0.81	1.88	0.69	2.13
Hold the items in mind and try to recall them backward from the last one first			1.81	1.94
Run through the list forward to the last item, and then repeat this for the item before the last one, and so on			1.19	0.44
Reverse the order of items (for example, in pairs or more of the items) as they are being presented			0.81	0.94
Reverse the order of items (for example, in pairs) just before they are recalled			1.00	0.56
Remember the last few items first and then do something else for the early items in the list			1.00	1.31

*Note.* Strategies and the frequency of their use in forward and backward recall in both the spatial and the digit recall tasks in Experiment 1. The scores are described in the text

### Results

#### Recall

The following analysis plan was adopted for this experiment. As the primary focus was on the response-time data, only data from trials in which all list items were correctly recalled were analyzed. Note that as participants have different spans, the response times for later positions are based on data from fewer participants. In order to compare adequacy of a peel-off strategy as an account of latencies, two simple models were fitted to the data for each participant in each of the two backward recall conditions. The first is a linear model where each item takes the same amount of time to recall. The second is the peel-off model. Both have two parameters corresponding to rate and intercept. In the linear model, rate is the inter-onset time for the recall of each item. Bayesian *t* tests were employed to test for group differences in the rate and intercept parameters. In the peel-off model, rate is the time to scan through each item in the forward phase of backward recall, and each successive item recalled requires one fewer item to be scanned than the previous one. Once the final item is recalled the estimate of the scanning rate will be identical to a full peel-off strategy. This will simply result in a lower (possibly negative) intercept as the intercept accounts for all time before the list-final item is output.

**Fig. 1:**
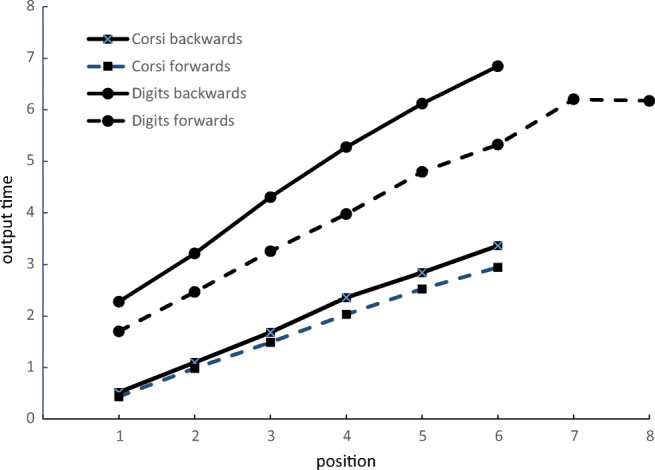
Mean output times (seconds) as a function of position for correctly recalled lists in Experiment 1

**Fig. 2:**
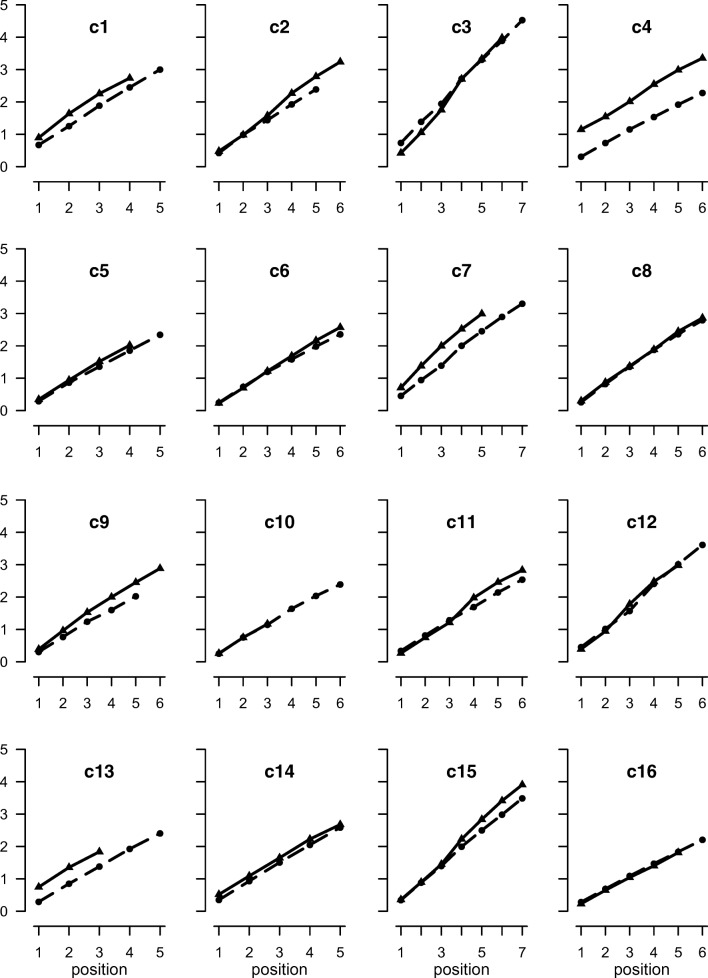
Mean output times (seconds) for spatial recall as a function of position for correctly recalled sequences for individual participants in Experiment 1. Solid lines are backward recall, dashed lines are forward

**Fig. 3:**
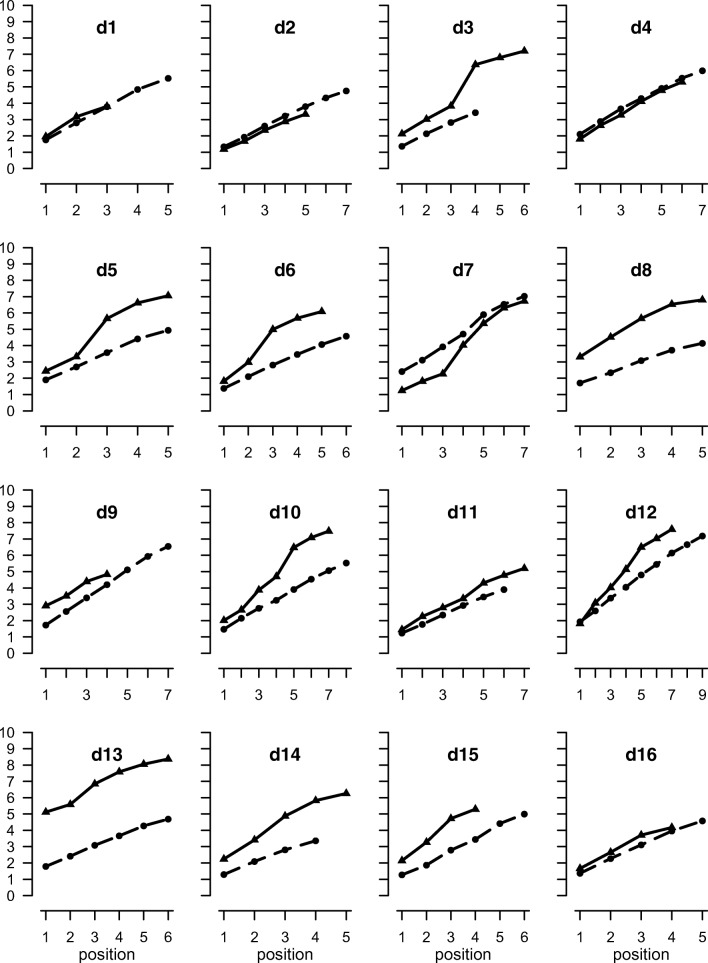
Mean output times (seconds) for digit recall as a function of position for correctly recalled lists for individual participants in Experiment 1. Solid lines are backward recall, dashed lines are forward

In unfamiliar tasks such as backward recall, it is quite plausible that a range of different strategies are adopted both within and across participants. This will result in considerable individual variability in recall latencies. We therefore report model statistics for both the group averages and for individual participants (see Appendix F). The adequacy of model fit as the group data was evaluated by comparing the *r*^2^ values of the two models. The response latency functions averaged across group are shown in Fig. 1, and their mean model parameters for the simple and peel-off models are shown in Table 2. The response time functions for individual participants are displayed in Figs. 2 and 3. For all measures reported here, the data for individual participants are given in the Appendices.

**Table 2:** Model fit statistics for Experiments 1 and 2A

Experiment	Stimuli	Model	Rate	Intercept	*r* ^2^
1	Visual digits	Forward linear	0.701	0.974	0.996
		Back linear	0.911	1.356	0.97
		Back peel-off	0.351	0.151	0.933
	Spatial locations	Forward linear	0.498	−0.101	0.998
		Back linear	0.546	−0.053	0.996
		Back peel-off	0.213	−0.726	0.961
2A	Spoken digits	Forward linear	0.493	0.193	0.998
		Back linear	0.829	0.886	0.976
		Back peel-off	0.293	−0.211	0.941

*Note.* Model fits to the timing data from Experiments 1 and 2a showing memory scanning rate (seconds/position), intercept (time), and *r*^2^ (proportion of variance accounted for). Forward and back linear are simple linear regression models. Back peel-off is the peel-off model described in the text

Strategy data were also collected as a secondary source of evidence for verifying the interpretation of the response time functions derived from the mathematical model-fitting procedure described above. Descriptive statistics allowed us to identify the profile of reported strategies for each individual and match them with their response-time functions. Specifically, it was expected that individuals with a negatively decelerating function expected of a simple peel-off strategy would report predominant use of this strategy.

##### Digit recall

Mean digit span at pretest was 6.1 for forward recall and 5.4 for backward recall. In the experimental trials, the mean proportion of lists correctly recalled was .57 forward and .63 backward. As shown in Fig. 1, the response time function across participants for forward digit recall is linear (slope = .701, mean *r*^2^ = .996). This pattern is present in all participants (Fig. 2). As shown in Table 2, the linear function also provided an excellent fit to the backward recall data (*r*^2^ = .970), although the mean intercept value was almost 400 ms higher than for forward recall and the slopes 200 ms greater. The fit of the peel-off function was good, although not as good as for backward recall (*r*^2^ = .933). There was considerably more variability in the patterns of response latencies in the backward than forward recall conditions (see Fig. 3). This is evident in the individual parameters shown in Appendix A, in which six of the 16 participants showed numerically greater *r*^2^ values for the peel-off than the simple linear model: d5, d6, d8, d13, d14, and d16. Participants generally took longer to output the first item in backward than forward recall (2.20 versus 1.63 seconds), with one participant (d13) slowing by more than 3 seconds.

Other more complex models could in principle have been constructed. One example is a three-parameter peel-off model with separate parameters for scanning rate and for executing the response to recall each item. However, there is little to be gained in explanatory power because the simple two-parameter models both produce excellent fits to the data and very similar timing functions. For example, a linear function fitted to a peel-off model with a backward span of five items has an *r*^2^ of .947. It is possible for both models to have *r*^2^ of greater than .98 when fitted to the same data. Not surprisingly then, there is very little difference between the fits of the two models.

##### Spatial recall

Mean forward span at pretest was 5.8 and backward span was 5.2. In the experimental trials the mean proportion of forward lists correctly recalled was .57 and for backward recall it was .59. Figure 1 shows that the slopes are approximately linear in both conditions. The linear functions yielded slopes of 498 ms for forward recall and 546 ms for backward recall, and *r*^2^ values of .998 for forward recall and .996 for backward recall. The forward and backward response times for each participant are shown in Fig. 3. The rate of recall appears to be linear in both directions for all participants.

#### Strategy reports

Table 1 summarizes the frequency of reported strategies across conditions. For this purpose, the four frequency descriptors of *never, occasionally, frequently,* and *almost always* were recoded as 0, 1, 2, and 3, respectively. Strategy ratings for each participant for each strategy and condition are reported in Appendices C–F.

For backward digit recall the most common strategies were rehearsal (2.25), grouping (2.0), backward scanning (1.81), peel-off (1.19), and reversal of input (0.81). In the corresponding spatial recall condition, the dominant recall strategies were forming an image (2.12), backward scanning (1.93), rehearsal (1.75), and forming a pattern (1.65). The mean score for peel-off was 0.44. Thus, contrary to what we might have expected from Anders and Lillyquist (1971), peel-off was not reported to be the most commonly used retrieval strategy for either of the backward recall conditions. The average rating for this strategy fell between *occasionally* and *almost never*.

In the previous section we reported a better fit to the response-time data for the peel-off model than a linear model for six of the 16 participants. Of these, peel-off was rated as being almost always used by one participant, d6 (see Appendix D). However, d6’s timing function looks almost identical to d5’s, who did not report ever using peel-off. For backward spatial recall, no participant reported more that occasional use of peel-off.

**Fig. 4:**
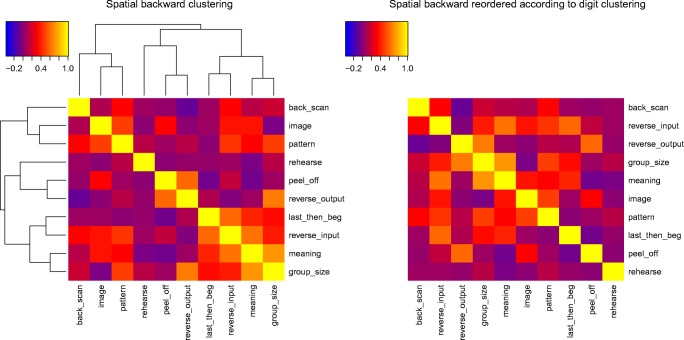
Figure 4a shows the intercorrelations between frequencies of strategy use for spatial recall. The clustering uses the agglomerative average clustering as implemented in the R function heatmap3. Figure 4b shows the same intercorrelations, but with rows and columns ordered in the same way as for the digit intercorrelations in Fig. 5

**Fig. 5:**
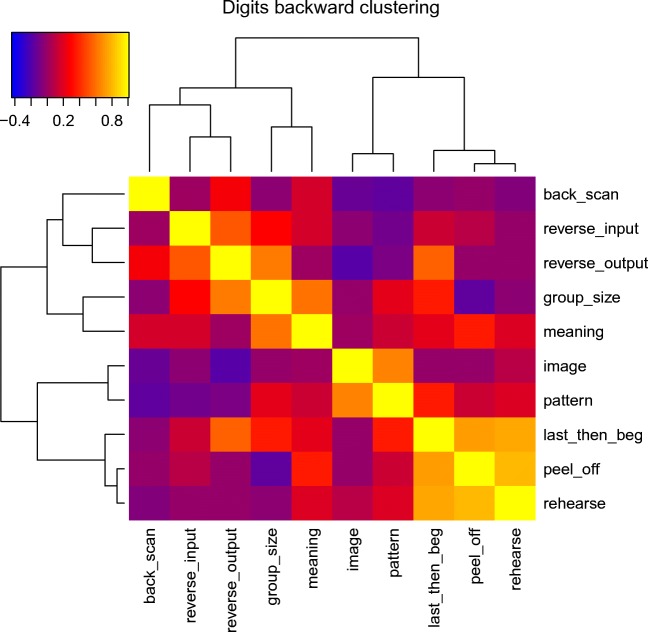
Intercorrelations between frequencies of strategy use for digit recall in Experiment 2a

Table 3 shows the correlations between the use of different strategies for both digit and spatial backward recall. Figures 4 and 5 show the correlations between strategy use ratings for backward recall, with the strategies clustered according to their correlations. Figures 4a–b show the strategy correlations for spatial recall when the strategies are ordered according to the digit strategy clustering, and the digits when ordered by the spatial clustering. This makes it easier to appreciate the relation between the two. For spatial recall, the strategies of backward scanning, forming an image, and forming a pattern constitute a cluster. So too do grouping by size, using meaning, reversing the input, and a strategy of last out then start at the beginning. In the case of digits, peeling off and last-then-begin formed the strongest cluster, along with rehearsal. This is interesting as it tells us that although peeling off alone was not a common strategy, it was often used in conjunction with recalling the last item(s) first. These two strategies are not mutually incompatible and could conceivably have been used in combination within a single trial. Thus, participants may have output the final (and probably, readily retrievable) item first and then turned to peel-off to support the output of the remaining list items. Note that the peel-off model can also provide a perfect fit in cases where the last item is output immediately rather than being recalled following a complete forward scan. These two strategies will differ only in intercept. That is, fitting the peel-off model to the case where the last item is recalled immediately will result in an earlier intercept.

**Table 3:** Correlations between strategy ratings for Experiment 1

		Strategy									
Condition: Strategy		Rehearse	Group by size	Group by pattern	Group by meaning	Mental image	Backward scan	Peel-off	Reverse at input	Reverse at output	Last out
Backward spatial recall											
	Rehearse	1									
	Group by size	0.026	1								
	Form pattern	0.033	0.279	1							
	Group by meaning	−.0.260	0.553	0.165	1						
	Form image	−0.124	−0.222	0.288	0.204	1					
	Backward scan	−0.019	0.066	0.159	0.032	0.015	1				
	Peel off	−0.118	−0.012	−0.045	−0.271	0.166	−0.077	1			
	Reverse at input	−0.015	0.226	0.274	0.416	0.198	0.168	0.046	1		
	Reverse at output	−.0.055	0.437	0.035	−0.030	−0.128	−0.365	0.399	−0.200	1	
	Last out	−0.091	0.185	−0.141	0.236	−0.051	−0.052	−0.297	0.370	0.013	1
Backward digit recall											
	Rehearse	1									
	Group by size	−0.118	1								
	Form pattern	0.103	0.107	1							
	Group by meaning	0.097	0.421	0.066	1						
	Form image	0.021	−0.054	0.479	−0.051	1					
	Backward scan	−0.207	−0.124	−0.400	0.085	−0.342	1				
	Peel-off	0.688	−0.387	0.063	0.218	−0.071	−0.075	1			
	Reverse at input	−0.080	0.166	−0.284	0.085	−0.112	−0.025	0.029	1		
	Reverse at output	−0.067	0.439	−0.244	0.000	−0.427	0.142	−0.055	0.330	1	
	Last out	0.645	0.210	0.219	0.115	-0.073	-0.113	0.593	0.056	0.359	1

*Note.* Intercorrelations between strategy ratings for both spatial and digit recall in Experiment 1

### Discussion

Response latencies in three of the four conditions in Experiment 1 were very well explained by a linear model in which the output time of each item was constant across all output positions. These were forward digit recall, and both forward and backward recall of spatial locations. This applied to all participants. These data are consistent with the operation of a retrieval strategy that has equivalent ease of access to the representation of each successive item to be output in these three conditions. For spatial recall, the equivalence of forward and backward recall in this respect points to a form of encoding which does not preferentially favor the forward-going direction of the original input sequences. In other words, backward serial recall appears to proceed in an equivalent sequential fashion from either end of the sequence.

For digit recall, the findings are less clear cut. The average fit of the linear model to the backward recall response-time data is very high, greater than that of the peel-off model in backward digit recall. However, the peel-off model provided a better fit for a third of the participants. Of these, only one reported frequently using peel-off. While it is of course possible that this strategy could be adopted without being available to introspection, this seems relatively implausible given the unusual and complex nature of the strategy, which is likely to require substantial attentional control. We lean toward the view that although this method of successive forward scanning is not the dominant strategy in backward verbal recall, it is spontaneously used by a small minority of participants.

The seeming rarity of use of a peel-off strategy for backward recall had not been anticipated given the findings reported by Anders and Lillyquist (1971) and Haberlandt et al. (2005). One possible explanation for the apparent disparity is that the pattern of linear functions of response times combined with delays in initiating backward recall attempts observed in Experiment 1 may have been a consequence of the mode of recall in this study. In the digit recall conditions this involved tapping on a soft keypad in an iPad. This method was chosen for two reasons: It provided parity with the tapping of spatial locations in the spatial recall test and was also convenient for automated scoring of the accuracy and timing of recall responses. On reflection, it seemed possible that the familiarity of the spatial layout of the keypad may have inadvertently encouraged recoding of the verbal sequences into the corresponding spatial layout immediately prior to recall. Such a strategy is much less likely with the spoken recall procedure employed by Anders and Lillyquist and Haberlandt et al. and so could the source of differences in prevalence of peel-off between the present and preceding response time studies of backward recall. An additional concern is that response modality may be a factor in determining how backward recall is performed. Beaudry, Saint-Aubin, Guérard, and Pâquet (2017) found that effects of word frequency and imageability were greatly reduced with spoken rather than manual recall.

Experiment 2 was conducted to investigate whether the recall method we used for the digit recall conditions had biased strategy choice in this way. In order to align the current method as closely as possible with that adopted in previous studies of response timing in forward and backward serial recall, Experiment 2 used spoken presentation and spoken recall. In order to provide a better estimate of individual variability in strategies, the sample size was increased to 24 participants.

## Experiment 2a

### Method

#### Participants

Participants were 24 volunteers from the Cognition and Brain Sciences Volunteer panel (mean age = 22.9 years, two males). Stimuli were presented on a laptop computer programmed in Python, and spoken responses were recorded directly onto the laptop using an AT2029 USB microphone. Response times were subsequently measured using the TotalRecall program (http://memory.psych.upenn.edu/AnnotationGuide). Stimulus presentation and timing were identical to those in the digit recall task in Experiment 1, except that the spoken recalls were marked online by the experimenter when setting span. The span criterion was three out of six lists correct. The order of recall was counterbalanced across participants, and there were 100 trials each for forward and backward recall.

#### Stimuli

The digits 1–9 and the words *ready* and *recall* were recorded by a male speaker using a high-quality microphone and sampling at 16 bits/44.1KHz. The resulting digit wav files were edited to be 1 s in length, with the location of the digit in the file adjusted so that sequences of digits would sound evenly paced no matter in which order they were played. Each digit list in the experiment was preceded by the word *ready* and followed by the word *recall*.

### Results and discussion

#### Recall

**Fig. 6:**
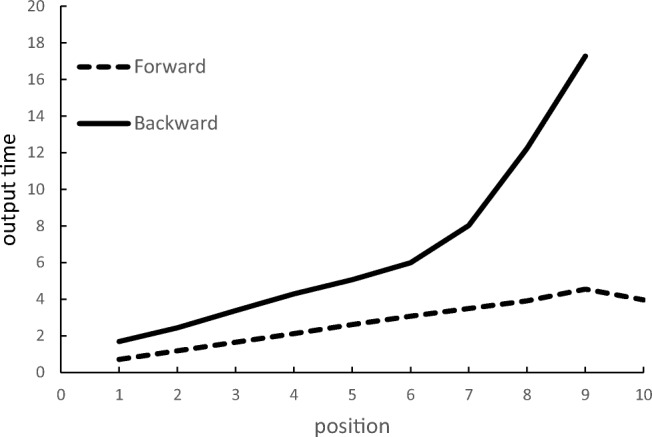
Mean output times (seconds) as a function of position for correctly recalled lists in 2a

**Fig. 7:**
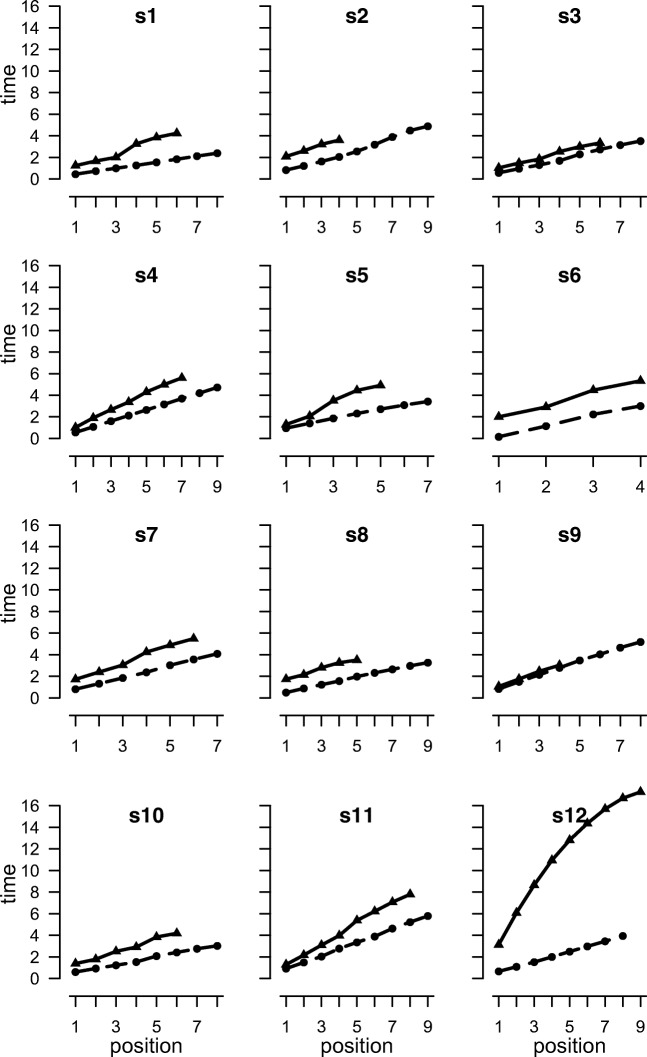

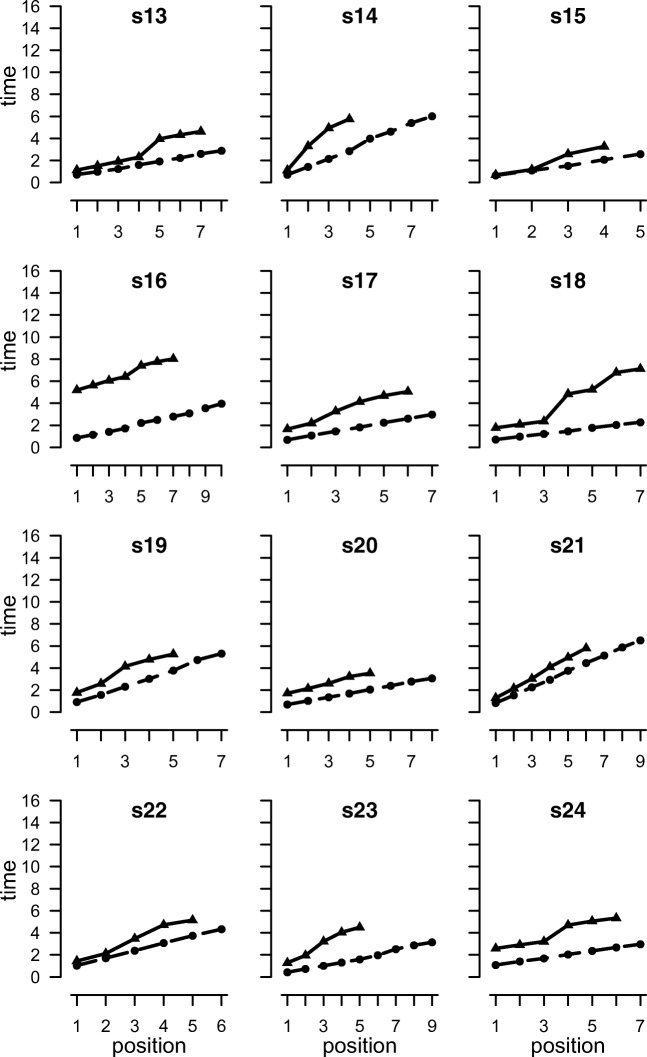
Mean output times (seconds) for digit recall as a function of position for correctly recalled lists for individual participants in 2a. Solid lines are backward recall, dashed lines are forward

Mean span was 7.7 for forward recall and 5.7 for backward recall. The proportion of lists correctly recalled was 0.61 forward and 0.72 backward. The mean onset times for responses in correctly recalled lists averaged over participants are shown in Fig. 6, and for individual participants in Fig. 7. There was individual variability in response time functions. The shape of the function for backward recall is strongly influenced by a single participant (s12) with a large backward span, whose performance therefore contributes disproportionately to later positions. The rate of forward recall is constant over positions, yielding a linear function. Backward recall is more variable and has a steeper slope and a higher intercept than forward recall (mean forward: slope = 0.493, intercept = 0.193, *r*^2^ = 0.998; backward: slope = 0.829, intercept = 0.886, *r*^2^ = 0.976). To examine the effect of recall order, we conducted Bayesian *t* tests^1^ to compare forward and backward recall. Recall order differences were found both in slope (BF_10_ = 2883) and intercept (BF_10_ = 13). Linear regressions established that the smallest *r*^2^ is .991 for forward recall and .933 for backward recall. The time to produce the first digit in backward recall was faster than for the keypad recall in Experiment 1 (1.69 vs. 2.20). This is consistent with the possibility that participants may not have done as much reordering immediately prior to commencing recall in Experiment 2 than Experiment 1.

The linear and peel-off models were fitted to the data for each participant. The response time functions for three participants (s12, s14, s19) were better fitted by a peel-off model than a simple linear function (see Appendix C), with *r*^2^ values for peel-off of .998, .999 and .973, respectively (see Appendix G). These are also the three best fits to the peel-off model. Further insights can be had from the rate estimates.

Any participant who predominantly used a peel-off strategy would be expected to show three characteristics. First, their data should be better fitted by the peel-off model than the linear model. Second, the estimate of the memory scanning rate derived from the peel-off model should be similar to the forward scanning rate, as both rates reflect the time to retrieve an item. Third, assuming reliable introspective access to how this unusual task of backward serial recall is accomplished, they should report predominantly using a peel-off strategy.

#### Strategy reports

**Fig. 8:**
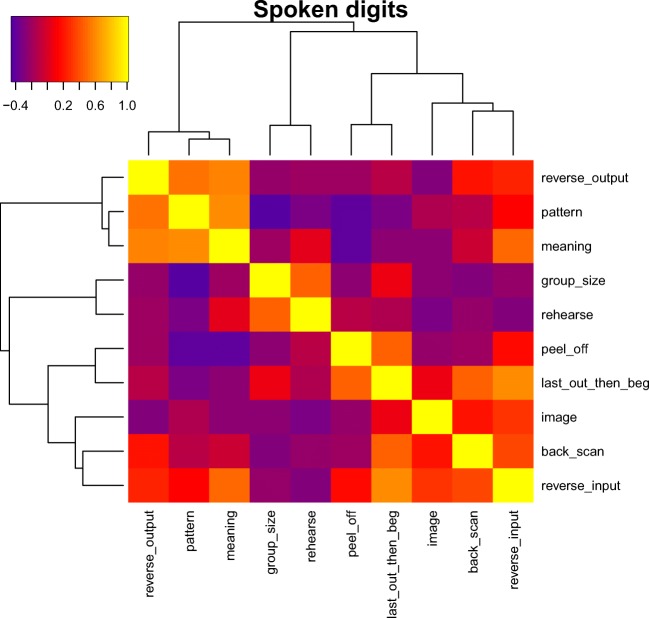
Intercorrelations between frequencies of strategy use for backward spoken digit recall in 2a

The strategy report data are summarized in Table 4. The dominant strategies for backward spoken recall were grouping by size (2.17), rehearsal (2.12), imagery (1.54), and backward scan (1.42). Peel-off was again one of the less frequently reported strategies (1.04), rated as being more commonly used only than meaning (0.67) and reversal at input (0.79). The reduced frequency of report of strategies involving forming an image and pattern compared with Experiment 1 seems likely to be a consequence of the move to spoken recall in the present experiment. Table 5 shows the correlations between strategies, and Fig. 8 presents the clustered correlations between the strategies.

**Table 4:** Self-reported strategy use in 2a as a function of recall direction

Strategy	Recall direction	
	Forward	Backward
Rehearse the items as they were presented	3.29	3.13
Group the items by separating them into sets of particular sizes	3.58	3.17
Group the items according to the pattern they form	2.04	2.04
Group the items according to their meaning	1.58	1.67
Form a mental image	2.37	2.54
Hold the items in mind and try to recall them backward from the last one first		2.42
Run through the list forward to the last item, and then repeat this for the item before the last one, and so on		2.04
Reverse the order of items (for example, in pairs or more of the items) as they are being presented		1.79
Reverse the order of items (for example, in pairs) just before they are recalled		2.33
Remember the last few items first, and then do something else for the early items in the list		2.29

*Note.* Self-reported strategies and the frequency of their use in forward and backward recall for spoken digit recall in 2a. The scores are described in the text

**Table 5:** Correlations between strategy ratings for 2a

Strategy	Strategy									
	Rehearse	Group by size	Group by pattern	Group by meaning	Mental image	Backward scan	Peel-off	Reverse at input	Reverse at output	Last out
Rehearse	1									
Group by size	0.337	1								
Form pattern	−0.258	−0.438	1							
Group by meaning	0.110	−0.029	0.533	1						
Form image	−0.252	−0.126	0.018	−0.138	1					
Backward scan	−0.083	−0.165	0.022	0.067	0.193	1				
Peel-off	0.033	−0.123	−0.389	−0.390	−0.089	−0.014	1			
Reverse at input	−0.161	−0.063	0.160	0.367	0.266	0.304	0.177	1		
Reverse at output	0.000	−0.071	0.426	0.506	−0.193	0.198	−0.047	0.233	1	
Last out	0.004	0.123	−0.244	−0.117	0.128	0.354	0.349	0.511	0.020	1

*Note.* Intercorrelations between strategy ratings for spoken digit recall in 2a

Of the three participants whose timing data were better fitted by the peel-off model (s12, s14, s19), only two (s12, s14) reported almost always using peel-off. One of them (s12) also reported almost always recalling the last item first (see Appendix I). As noted earlier, these two strategies are entirely compatible if participants immediately recall the last item first and then peel off the remaining items. Only one other participant reported almost always using peel-off (s20), but this individual reported almost always using all four possible recall strategies. Furthermore, their timing data were better fitted by the linear model than by peel-off (.992, .948, respectively). The final individual (s19) reported never using peel-off.

If we base inferences about strategies on the concordance of evidence from model fits and strategy reports, only s12 and s14 therefore appear to be using peel-off. This conclusion is also consistent with the rate parameters. Participant s12 had by far the slowest rate of recall, taking about 17 s to recall nine items. However, the rate derived from the peel-off model was very similar to that from forward recall (peel-off: .388, forward: .471). Less convincingly, the rate estimated from the linear model of backward recall (1.765) was 3.7 times slower than the estimate from forward recall.

Participant s14 shows a similar pattern. The estimated scanning rate from the peel-off model is almost identical to the rate derived from forward recall (.774 and .781), and the rate estimated from the linear model is also very slow (1.558)—more than 0.5 s slower than the next slowest participant. Only s12 and s14 therefore show all three signs of using peel-off. No other participant showed more than one.

The participants who did not report high levels of use of peel-off exhibited a range of response-time functions which are not readily associated with specific strategies. Several participants seem to show a discontinuity in the middle of the list (s1, s13, s16, s18, s19, s24). It seems likely that this reflects a grouping strategy with a retrieval delay between recall of the last item of the first group and the first item of the next. This would be consistent with the strategy for backward recall suggested by Guerrette, Guérard, and Saint-Aubin (2017) and by Anderson et al. (1998).

We applied the model-fitting approach employed in the present experiment to the data from Anders and Lillyquist’s participant who reported using the peel-off strategy. The data were extracted from their Fig. 2 using WebPlotDigitizer (https://automeris.io/WebPlotDigitizer). This figure plots cumulative interword pause times. The mean duration of each spoken digit (over all participants) was .197 s. We can therefore use the data from the figure to estimate word onset times by adding the summed duration of previous words to each of the times extracted from the figure. The linear model (*r*^2^ = .98) fits the data better than the peel-off model (*r*^2^ = .94). The data from Fig. 2c of Haberlandt et al. (2005) were also extracted. For these data, too, the linear model produced a very good fit to the model at all list lengths (all *r*^2^ > .99).

## Experiment 2b

### Method

We have proposed on the basis of converging strategy reports and retrieval rates that the negatively decelerating response time function exhibited by s12 and s14 is indeed a hallmark of peeling off. To assess this more directly, we tested a further group of participants to establish whether instruction to use a peel-off strategy in backward recall is sufficient to induce this response-time function. These participants first completed a set of backward recall trials under the standard condition of no strategy instruction employed in the main experiment. They then completed further set of trials following explicit instruction to use a peel-off strategy. Our reasoning was that as long as participants were able successfully implement the peel-off strategy, they should produce a function similar to s12.

Four participants took part in this extension to Experiment 2a. They each completed two sets of backward recall 100 trials. In the first set of trials, there were no strategy instructions as in Experiments 1 and 2a. Immediately prior to the second set of trials, the experimenter instructed participants to use the peel-off strategy. Participants were told that they should “run through the list forward to the last item, recall, then repeat for the item before the last one, and so on.” The experimenter then gave a demonstration of the strategy by saying the recall procedure aloud. Span was reassessed immediately after giving the explicit strategy instructions, setting the list length for the following experimental trials. The mean age of the participants was 26.7 years (one male).

### Results and discussion

**Fig. 9:**
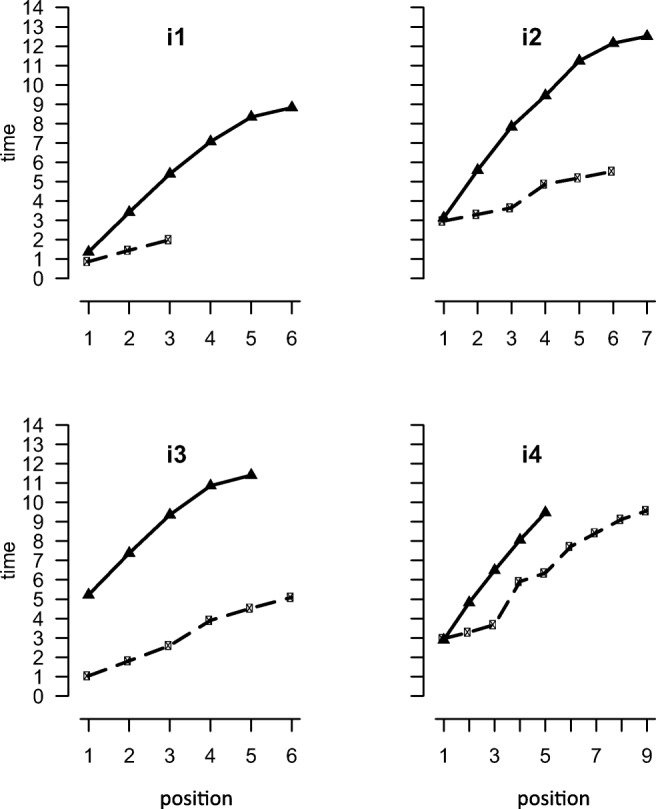
Mean output times (seconds) for backward digit recall as a function of position for correctly recalled lists for the participants given instructions in Experiment 2b. Solid lines are for recall given peel-off instructions, dashed lines are for recall with no instructions

The results are shown in Fig. 9 and the statistics for the linear and peel-off models for each of the free and instructed trial blocks are displayed in Table 6. When participants were not instructed to adopt the peel-off strategy, they did not show the negatively accelerated function that we have taken to be a reflection of peel-off. Recall was slower when participants were instructed to adopt the peel-off strategy. For i1, i2, and i3, the output times have the negatively accelerated form expected on the basis of peel-off. Individual fits to the peel-off model are very good, with only participant (i4) having a better fit to the linear model than to the peel-off model. Further evidence that at least three that participants are using peel-off is provided by comparing the estimates of scanning rate (slope values) for the two alternative models. For forward recall, the rate was approximately 0.5 s per item. A similar value (mean = .553) was obtained when the peel-off model was fitted to these four participants. For the linear model, the rate is approximately 1.59 s. Thus, if we assume that participants are able to scan through the list backwards, then the scanning time is roughly 3 times slower than when scanning forwards. However, when we can be confident that participants are performing peel-off, the rate of scanning seems to be similar to what would be predicted from measuring forward recall.

**Table 6:** Model parameters for each participant in 2b

Participant	Condition	Model	Rate	Intercept	*r* ^2^
i101	Free	Linear	0.565	0.313	1.000
		Peel-off	0.364	−0.257	0.995
	Instructed	Linear	1.536	0.362	0.966
		Peel-off	0.505	−1.917	0.995
i102	Free	Linear	0.564	2.280	0.954
		Peel-off	0.178	1.559	0.998
	Instructed	Linear	1.595	2.464	0.953
		Peel-off	0.453	−0.205	0.998
i103	Free	Linear	0.851	0.189	0.987
		Peel-off	0.271	−0.949	0.996
	Instructed	Linear	1.585	4.089	0.960
		Peel-off	0.628	1.933	0.996
i104	Free	Linear	0.920	1.737	0.970
		Peel-off	0.195	0.182	0.970
	Instructed	Linear	1.639	1.430	0.997
		Peel-off	0.629	−0.574	0.970

*Note.* Model fits to the timing data from participants performing backward recall in 2b. Table shows memory scanning rate (seconds/position), intercept (seconds), and *r*^2^ (proportion of variance accounted for) by the linear model and the peel-off model according to whether participants were free to use their own strategy (first half of experiment) or instructed to use the peel-off strategy (second half of experiment)

## General discussion

Two experiments investigated whether backward recall is performed by applying a peel-off strategy that involves successive forward scans to pick off the last item in a sequence first, then the penultimate one, and so on. On the basis of the data from backward recall for verbal sequences reported by Anders and Lillyquist (1971) and Anderson et al. (1998), we expected to find that the dominant strategy for performing backward verbal recall would involve using successive forward recalls. Forward and backward recall of spatial sequences were also included in the first experiment in order to establish whether the peel-off strategy is a consequence of forward-going representations of serial order that is restricted to verbal stimuli (Hurlstone et al., 2014). On the basis of the near-equivalence in the accuracy of forward and backward spatial recall (Isaacs & Vargha-Khadem, 1989; Wilde & Strauss, 2002), it seemed at least possible spatial representations are retrieved as readily in the backward as the forward direction. If this was the case, there should be no evidence for a peel-off strategy in backward spatial recall.

Contrary to expectations, the findings provide little evidence that a peel-off strategy is used spontaneously in the backward recall of either verbal or spatial sequences. Of the 40 participants performing backward digit recall in Experiments 1 and 2a, just three (d6, s12, s14) showed what we take to be the signature of the peel-off strategy: a negatively accelerating response-time function reflecting the reducing number of items to be scanned in the forward direction as recall progresses, combined with self-report that this strategy was almost always used. In backward spatial recall (Experiment 1) as in all forward recall conditions across both experiments, linear response time functions were observed.

In Experiment 1, participants recalled the sequences by tapping screen locations corresponding to either digits on a 3 × 3 telephone keypad layout or circles corresponding to the spatial locations. Evidence for peel-off was found for just one participant. One possibility is that the apparent failure to adopt a peel-off strategy for verbal material here may have reflected spatial recoding of the keypad locations of the digit responses. In contrast, the benchmark study by Anders and Lillyquist (1971) employed spoken verbal recall. This possibility was ruled out in Experiment 2a. With spoken recall rather than keypad selection of responses, there was evidence of a peel-off strategy for backward recall in only two participants. Our confidence that the negatively accelerated response-time function observed in the backward digit recall condition for two of the participants in Experiment 2a reflects that the peel-off strategy was reinforced by data from a further four participants in Experiment 2b, who completed backward digit recall first with no strategy guidance, as in the previous experiments, and, second, following instructions to use a peel-off strategy. Three of the participants (i1, i2, i3) showed the predicted negatively accelerated response-time function following strategy instruction. This was not evident when participants were free to adopt their own strategies, but emerged following strategy instruction. More tellingly, when instructed to perform peel-off, the estimated rate of memory scanning according to the peel-off model was similar to the rates of forward recall in 2a. We do not know whether the remaining participant really did successfully implement the peel-off strategy.

We had originally anticipated that careful analysis of the timing data would allow us to differentiate between participants adopting different strategies that could then be validated against their strategy reports. In fact, it turned out that most of the timing functions were well fitted by both the linear and the peel-off models, providing little room for discriminating between the two. Indeed, the same functions would apply for most other commonly reported strategies, such as reversing pairs of items. An important discovery that we did make was that most participants reported using multiple strategies. Just three reported using only a single recall strategy. If these introspective reports are reliable, this means that average response times will necessarily be driven by a combination of strategies and not by a single one. Response time functions are only capable of discriminating between strategies if the strategies are consistently applied and the predicted functions are quite distinct. If people use a range of strategies, then the response-time function across all trials is likely to favor the simple linear model over more complex models. Although the model fits provided little basis for discriminating between strategies, the value of the fitted rate parameters was far more diagnostic. If participants perform peel-off, then we would expect their scanning rate to be similar for forward and backward recall, and this was indeed the case.

Our conclusion is therefore that people do not usually perform backward verbal recall in the way that has been widely assumed. Many different strategies are employed and, occasionally, these include the successive forward retrievals involved in the peel-off strategy previously assumed (Anders & Lilliquist, 1971; Conrad, 1965). The suggestion that people do use a peel-off strategy may have simply arisen from assumptions about the way that order is stored in STM. In the case of a simple slot model (Conrad, 1965), it might be assumed that information could be read out of memory equally well in forward or backward direction. The spatial recall data in Experiment 1 are broadly consistent with this account. However, even in Experiment 1, where there was little difference in span between forward and backward digit recall, backward recall was much slower. This suggests that it is more challenging. Other differences between forward and backward recall indicate that they differ in terms of more than direction. For example, Bireta et al. (2010) found that backward recall eliminated or attenuated the effects of word length, irrelevant speech, phonological confusability, and articulatory suppression. The implication is that either backward recall does not depend on the use of the same storage system that underlies forward recall, or that it uses STM in a substantially different way from a standard serial recall procedure.

In contrast to a slot model, most computational models of verbal STM are intrinsically directional. For example, in the primacy model (Page & Norris, 1998), recall must proceed in a forward direction by successively recalling the most strongly activated item. Indeed, Page and Norris (1998) modeled backward recall as a peel-off process. Other models either also rely on some form of primacy gradient or on some form of evolving context that becomes associated with successive items, and whose forward evolution can be reconstructed (Brown, Preece, & Hulme, 2000; Burgess & Hitch, 1992, 1999; Farrell & Lewandowsky, 2002; Lewandowsky & Murdock, 1989). None of these models has a ready account of how backward recall might be performed. The fact that backward recall does not need to involve peel-off implies that there is far more flexibility in the readout of serial order than the standard computational models would imply. A further challenge for models of STM is the fact that, in contrast to verbal recall, spatial recall seems to be performed in the same way in both directions.

One of the reasons why psychologists are interested in backward recall is its widespread use as an index of cognitive capacities critical for general cognitive outcomes across the life span. We have learned from this study that there is no single cognitive strategy that characterizes backward span, and also that the majority of people, left to their own devices, will try out a multiplicity of strategies, often in combination. Although peeling off items in successive forward retrievals may be effective, in the present experiments it was used only rarely. This may be due to the large number of cognitive operations it requires, making it slow and resource demanding. The data indicate that the use of this strategy does not necessarily improve performance. Of the three participants who reported predominantly using it in 2a, one had the highest backward span of all participants, but the spans of the other two were unremarkable. Moreover, in 2b, backward span improved for only one of the three participants who appeared to be using peel-off when instructed to do so.

Perhaps it is this very variability in the strategies adopted by individuals that makes backward recall such a good predictor of outcomes such as academic achievement and cognitive ageing. The sophistication and optimality of the strategies adopted must be weighed against their processing costs as a function of the total cognitive resources available, which will inevitably vary across individuals. A complex strategy such as peel-off may only benefit individuals with exceptional cognitive resources whereas for others, the costs may outweigh the mnemonic benefits. Despite its simplicity, the selection and efficacy of execution of strategies in backward recall may therefore provide an excellent index of both the cognitive resources and cognitive flexibility that an individual can bring to bear in novel and cognitively challenging situations.

## Appendix A

**Table 7:** Experiment 1. Parameters for backward digits

Participant	Linear rate	Linear intercept	Peel-off rate	Peel-off intercept	Linear *r*^2^	Peel-off *r*^2^
d1	0.927	1.126	0.616	0.103	0.968	1.000
d2	0.549	0.635	0.210	−0.024	0.996	0.958
d3	1.121	0.964	0.361	−0.583	0.939	0.927
d4	0.706	1.183	0.225	0.240	0.995	0.964
d5	1.253	1.259	0.491	−0.389	0.943	0.958
d6	1.123	0.941	0.447	−0.606	0.937	0.979
d7	1.018	−0.109	0.273	−1.493	0.970	0.906
d8	0.900	2.666	0.357	1.438	0.957	0.995
d9	0.665	2.247	0.321	1.505	0.985	0.962
d10	0.996	0.912	0.271	−0.531	0.977	0.943
d11	0.637	0.898	0.174	−0.026	0.991	0.956
d12	0.990	1.069	0.273	−0.440	0.983	0.976
d13	0.698	4.483	0.227	3.474	0.960	0.972
d14	1.045	1.386	0.411	0.003	0.969	0.988
d15	1.091	1.129	0.533	−0.138	0.976	0.977
d16	0.859	0.902	0.422	−0.114	0.976	0.987

## Appendix B

**Table 8:** Experiment 1. Parameters for backward spatial recall

Participant	Linear rate	Linear intercept	Peel-off rate	Peel-off intercept	Linear *r*^2^	Peel-off *r*^2^
c17	0.615	0.344	0.299	−0.362	0.991	0.985
c18	0.569	−0.103	0.180	−0.843	0.996	0.953
c19	0.730	−0.348	0.230	−1.285	0.996	0.945
c20	0.453	0.678	0.143	0.092	0.997	0.948
c21	0.559	−0.194	0.269	−0.812	0.998	0.967
c22	0.474	−0.229	0.150	−0.845	0.999	0.955
c23	0.570	0.209	0.220	−0.496	0.994	0.975
c24	0.516	−0.180	0.164	−0.858	0.999	0.956
c25	0.498	−0.041	0.159	−0.708	0.996	0.969
c26	0.453	−0.181	0.293	−0.644	0.998	0.978
c27	0.535	−0.289	0.169	−0.986	0.992	0.947
c28	0.671	−0.298	0.256	−1.105	0.993	0.954
c29	0.546	0.220	0.355	−0.346	0.996	0.985
c30	0.546	−0.006	0.208	−0.660	0.998	0.961
c31	0.610	−0.284	0.165	−1.139	0.997	0.947
c32	0.392	−0.150	0.149	−0.612	0.999	0.952

## Appendix C

**Table 9:** Experiment 1. Strategy data for forward digit recall

	Strategy					
Participant	Rehearse	Group sizes	Group pattern	Group meaning	Mental image	Span
d1	3	2	0	0	0	5
d2	1	3	2	0	3	7
d3	3	0	0	1	1	4
d4	1	3	1	0	0	7
d5	3	3	0	1	0	5
d6	3	1	0	0	1	6
d7	3	3	1	0	0	7
d8	3	1	0	0	0	5
d9	2	3	0	0	0	7
d10	1	3	0	0	0	8
d11	3	2	0	1	1	6
d12	3	3	1	0	0	9
d13	3	3	1	0	1	6
d14	2	1	2	1	3	4
d15	3	3	0	0	1	6
d16	2	2	0	0	2	5

## Appendix D

**Table 10:** Experiment 1. Strategy data for backward digit recall

Participant	Strategy										
	Rehearse	Group sizes	Group pattern	Group meaning	Mental image	Backward scan	Peel-off	Reverse on input	Reverse then output	Last then do something else	Span
d1	3	0	0	0	0	3	2	1	1	1	3
d2	1	3	2	0	2	1	0	0	0	1	5
d3	3	3	1	1	1	0	2	3	1	1	6
d4	1	1	0	0	0	2	0	3	1	0	6
d5	2	3	0	1	0	3	0	0	1	0	5
d6	3	0	0	0	1	0	3	0	0	1	5
d7	3	3	1	1	0	1	2	0	3	3	7
d8	3	2	3	0	0	1	2	0	1	2	5
d9	1	3	0	0	0	1	0	2	2	0	4
d10	1	3	0	0	0	3	0	0	3	0	7
d11	2	3	1	2	1	3	2	1	0	1	7
d12	3	2	0	0	0	3	1	0	0	1	7
d13	3	3	1	0	2	0	0	0	0	1	6
d14	2	0	2	0	3	2	1	0	0	0	5
d15	3	3	0	0	1	3	2	3	3	3	4
d16	2	0	0	0	0	3	2	0	0	1	4

## Appendix E

**Table 11:** Experiment 1. Strategy use in forward spatial recall

Participant	Strategy					
	Rehearse	Group sizes	Group pattern	Group meaning	Mental image	Span
c17	3	0	2	2	3	5
c18	2	0	1	0	2	5
c19	1	3	1	0	1	7
c20	3	2	2	0	1	6
c21	1	0	2	0	3	5
c22	1	3	3	2	1	6
c23	1	3	3	2	1	7
c24	3	0	3	0	2	6
c25	1	2	3	0	1	6
c26	1	3	2	0	2	5
c27	1	1	2	0	3	6
c28	2	0	1	0	0	6
c29	3	0	2	0	3	6
c30	3	0	1	0	2	5
c31	2	1	3	0	3	5
c32	3	1	3	0	2	7

## Appendix F

**Table 12:** Experiment 1. Strategy use in backward spatial recall

	Strategy										
Participant	Rehearse	Group sizes	Group pattern	Group meaning	Mental image	Backward scan	Peel-off	Reverse on input	Reverse then output	Last then something else	Span
c17	3	0	2	0	3	3	1	0	0	0	4
c18	1	0	1	0	2	2	0	1	0	2	6
c19	2	3	2	0	2	1	1	0	3	2	6
c20	3	2	1	0	1	2	0	0	1	0	6
c21	3	0	2	0	2	1	0	0	0	0	4
c22	1	2	3	1	3	1	0	2	1	2	6
c23	1	3	1	1	2	3	0	2	0	2	5
c24	1	0	2	0	2	3	0	0	1	0	5
c25	1	0	0	0	2	2	1	1	1	3	6
c26	0	1	2	0	2	2	1	1	0	0	6
c27	2	1	2	0	3	2	2	2	1	0	3
c28	1	0	1	0	1	1	1	1	1	1	6
c29	3	0	2	0	3	2	0	3	0	2	5
c30	3	0	0	0	2	1	0	0	0	2	3
c31	0	0	2	0	3	2	0	0	0	2	5
c32	3	2	3	0	1	3	0	2	0	3	7

## Appendix G

**Table 13:** 2a. Parameters for backward digits with spoken recall

Participant	Linear rate	Linear intercept	Peel-off rate	Peel-off intercept	Linear *r*^2^	Peel-off *r*^2^
s1	0.651	0.434	0.203	−0.371	0.966	0.898
s2	0.515	1.593	0.248	1.018	0.993	0.970
s3	0.480	0.527	0.151	−0.087	0.992	0.939
s4	0.770	0.323	0.209	−0.780	0.998	0.956
s5	0.963	0.355	0.373	−0.862	0.974	0.967
s6	1.155	0.792	0.551	−0.454	0.986	0.943
s7	0.786	0.873	0.248	−0.142	0.990	0.940
s8	0.465	1.290	0.180	0.703	0.980	0.970
s9	0.661	0.417	0.318	−0.313	0.997	0.968
s10	0.588	0.713	0.184	−0.017	0.986	0.921
s11	0.962	0.296	0.228	−1.182	0.995	0.948
s12	1.765	2.913	0.388	−0.561	0.958	0.998
s13	0.650	0.226	0.172	−0.618	0.943	0.861
s14	1.558	−0.114	0.774	−2.027	0.963	0.999
s15	0.914	−0.353	0.430	−1.292	0.966	0.899
s16	0.505	4.620	0.136	3.925	0.977	0.920
s17	0.725	0.961	0.234	−0.048	0.977	0.971
s18	1.012	0.261	0.269	−1.075	0.943	0.869
s19	0.917	0.956	0.359	−0.247	0.960	0.973
s20	0.472	1.221	0.180	0.662	0.992	0.948
s21	0.915	0.349	0.288	−0.819	0.999	0.945
s22	1.001	0.376	0.384	−0.843	0.976	0.947
s23	0.853	0.438	0.330	−0.630	0.977	0.964
s24	0.620	1.794	0.195	1.004	0.933	0.880

## Appendix H

**Table 14:** 2a. Strategy use in forward digits with spoken recall

Participant	Rehearse	Group sizes	Group pattern	Group meaning	Mental image	Span
s1	2	3	0	0	2	8
s2	1	1	2	0	2	9
s3	3	3	0	1	0	8
s4	3	3	2	1	2	9
s5	1	3	1	2	0	7
s6	1	0	1	1	0	4
s7	1	3	1	0	1	7
s8	2	3	2	0	0	9
s9	2	3	2	1	2	8
s10	3	3	1	0	3	8
s11	3	3	2	1	0	9
s12	2	3	0	0	0	8
s13	2	3	1	1	0	8
s14	2	2	1	0	2	8
s15	3	2	1	0	3	5
s16	3	3	1	1	2	10
s17	3	3	0	0	3	7
s18	2	3	0	0	2	7
s19	2	3	1	1	1	7
s20	2	3	1	1	1	8
s21	3	3	0	1	2	9
s22	3	0	2	0	2	6
s23	3	3	1	0	3	9
s24	3	3	2	2	0	7

## Appendix I

**Table 15:** Experiment 2a. Strategy use in backward digits with spoken recall

Participant	Strategy										
	Rehearse	Group sizes	Group pattern	Group meaning	Mental image	Backward scan	Peel-off	Reverse on input	Reverse then output	Last then something else	Span
s1	3	3	0	0	1	2	2	2	1	3	6
s2	2	0	1	0	2	1	2	0	1	0	4
s3	3	3	1	1	0	3	1	0	0	0	6
s4	3	3	1	0	2	1	1	0	0	1	7
s5	0	3	0	0	2	2	1	1	1	2	5
s6	2	0	2	2	0	3	0	0	3	0	4
s7	0	3	0	0	2	1	1	0	0	0	6
s8	1	0	3	0	2	0	0	0	0	0	5
s9	2	2	1	1	2	2	1	2	2	1	4
s10	3	3	1	2	3	1	1	3	1	3	6
s11	3	3	2	1	0	0	0	0	2	0	8
s12	2	3	0	0	0	0	3	0	0	3	9
s13	0	2	3	1	3	2	0	1	3	2	7
s14	2	0	0	0	1	0	3	0	1	1	4
s15	2	0	1	0	3	3	3	2	1	3	4
s16	3	3	1	1	2	0	0	0	1	0	7
s17	3	3	0	0	0	2	0	0	2	3	6
s18	3	3	1	0	2	2	2	0	3	0	7
s19	2	3	1	1	1	0	0	2	1	0	5
s20	2	3	1	1	1	2	3	3	3	3	5
s21	3	3	0	1	3	1	0	0	1	0	6
s22	1	0	3	2	2	3	0	3	2	2	5
s23	3	3	0	0	3	3	0	0	0	3	5
s24	3	3	2	2	0	0	1	0	3	1	6

## Footnotes

1 Bayes factors were calculated in R using the function ttestBF in Bayes Factor package, Version 9.12-2.
